# Beyond mechanical revascularization: thrombo-inflammatory mechanisms and vascular bed–specific design of intracranial stents

**DOI:** 10.3389/fmolb.2026.1785666

**Published:** 2026-04-28

**Authors:** Yingjun Liu, Jinquan Li, Liuxun Hu, Junzhe Zhu, Qingzhu An, Guo Yu

**Affiliations:** 1 Department of Neurosurgery, Huashan Hospital, Shanghai Medical College, Fudan University, Shanghai, China; 2 National Center for Neurological Disorders, Shanghai, China; 3 Shanghai Key Laboratory of Brain Function and Restoration and Neural Regeneration, Shanghai, China; 4 Neurosurgical Institute of Fudan University, Shanghai, China; 5 Shanghai Clinical Medical Center of Neurosurgery, Shanghai, China

**Keywords:** cerebrovascular disease, endothelial cells, inflammation, in-stent restenosis, stent implantation, vascular smooth muscle cells

## Abstract

Cerebrovascular and coronary artery diseases are among the leading causes of mortality. Stent systems are widely used in both cardiac and cerebrovascular interventions; however, in-stent restenosis and acute stent thrombosis associated with bare-metal stents can cause severe ischemic complications and markedly reduce therapeutic efficacy. Although drug-eluting stents significantly decrease the incidence of restenosis, they carry an increased risk of late and very late stent thrombosis. Currently, antiplatelet therapy remains the primary strategy to prevent stent-related thrombosis. Nevertheless, drugs eluted from stents can impair endothelial recovery. This delay in reendothelialization increases the risk of late thrombotic events. Therefore, a comprehensive understanding of the mechanisms underlying stent implantation–associated pathology is essential for the development of next-generation stent platforms and targeted pharmacological strategies. In this review, we systematically discuss the pathological mechanisms underlying stent-associated complications from four key perspectives: endothelial cells, vascular smooth muscle cells, the coagulation system, and inflammatory responses. By integrating current evidence from both coronary and intracranial vascular systems, this review aims to provide mechanistic insights to support the optimization of stent design and improve long-term clinical outcomes.

## 1 Introduction

Intracranial aneurysm (IA) is a cerebrovascular disease associated with high mortality and disability rates, with rupture-related morbidity and mortality rates reaching up to 60% (Nieuwkamp et al., 2009). In recent years, landmark trials, including the International Subarachnoid Aneurysm Trial (Molyneux et al., 2002; Molyneux et al., 2005) and the International Study of Unruptured Intracranial Aneurysms (Wiebers et al., 2003; International Study of Unruptured Intracranial Aneurysms I, 1998), demonstrated that endovascular therapy confers significant advantages over conventional open surgery in reducing both mortality and long-term disability (Marshman et al., 2007). Consequently, endovascular intervention has progressively become the dominant therapeutic strategy for cerebrovascular diseases, increasingly replacing open surgical approaches in clinical practice.

Despite continuous advances in medical technology and substantial improvements in survival among patients with coronary artery disease, cardiovascular diseases remain the leading cause of death globally. Epidemiological data indicate that cardiovascular disorders are responsible for approximately 17.9 million deaths annually, with projections exceeding 23.6 million deaths by 2030 (Smith et al., 2012; Laslett et al., 2012; Yahagi et al., 2016; Thomas et al., 2018; Williams et al., 2019).

Stent implantation has become a fundamental endovascular technique in the management of both cardiovascular and cerebrovascular diseases, markedly enhancing patient survival (Wang et al., 2025). However, in-stent restenosis (ISR) and stent-associated thrombosis persist as significant challenges after stent implantation, undermining long-term clinical outcomes (Wang et al., 2025; Trepanier et al., 2023). Drug-eluting stents (DES) were introduced to address these limitations. Although first-generation drug-eluting stents effectively reduced the incidence of ISR, they increased the risk of late and very late stent thrombosis. Therefore, elucidating the pathological mechanisms underlying ISR and thrombosis is essential for optimizing stent design and minimizing stent-related complications.

The pathological features of ISR exhibit marked heterogeneity across different vascular beds. Following stent implantation, disruption of the endothelial layer exposes the subendothelial matrix, triggering platelet adhesion and aggregation and activating the coagulation cascade, thereby increasing the risk of early thrombus formation (Todorov et al., 2021). In parallel, injured endothelial cells and exposed collagen rapidly initiate local inflammatory responses by releasing proinflammatory cytokines and chemokines, which recruit inflammatory cells such as monocytes and macrophages to the site of injury (Todorov et al., 2021; Guo et al., 2024). This inflammatory microenvironment profoundly regulates vascular smooth muscle cell (VSMC) behavior, promoting a phenotypic switch toward a proinflammatory state and contributing to neointimal formation (Todorov et al., 2021). In the context of atherosclerosis, the pathological microenvironment at the stent implantation site becomes increasingly complex. Oxidative stress, hyperlipidemia, and chronic inflammation further exacerbate endothelial dysfunction and abnormal VSMC responses, thereby facilitating the development of late ISR (Liu L. et al., 2023).

Importantly, dysregulation of the coagulation system persists throughout the entire process. However, coagulation activation and inflammatory signaling are tightly interconnected and operate as a self-amplifying thrombo-inflammatory circuit rather than independent pathways. Following endothelial injury, von Willebrand factor (vWF) plays a pivotal role in early thrombus formation (Todorov et al., 2021). Antiproliferative agents, such as sirolimus and paclitaxel, effectively inhibit VSMC proliferation and neointimal hyperplasia; however, their effects on endothelial repair and the risk of late thrombosis remain incompletely understood (Park et al., 2025). Collectively, these findings indicate that ISR and thrombosis should not be regarded as isolated pathological events, but rather as interconnected processes driven by complex molecular and cellular interactions, forming a dynamic pathological network (Xiao et al., 2025; Rao et al., 2025). A deeper understanding of this network is critical for the development of next-generation stent-based therapeutic strategies.

## 2 Clinical evolution and persistent challenges of stent therapy

The application of stent systems in both cerebrovascular and cardiovascular diseases has undergone substantial expansion over the past 2 decades. In the intracranial circulation, representative devices include flow-diverting stents characterized by dense mesh structures (Zhou et al., 2014) and covered stents such as the Willis stent (Li et al., 2007; Wang et al., 2008). Beyond cerebrovascular disorders, stent-based therapies are widely applied throughout the systemic circulation, particularly in coronary artery disease and atherosclerotic conditions (Duffy et al., 2015; Aristokleous et al., 2016). Among these indications, coronary stenting remains the most extensively studied and clinically implemented endovascular intervention.

Despite remarkable technological progress, long-term outcomes after stent implantation continue to be limited by in-stent restenosis (ISR) and stent-associated thrombosis. These complications persist across vascular territories and represent the principal determinants of durability and safety. In coronary practice, antiproliferative drug-eluting platforms were developed to mitigate excessive neointimal hyperplasia (Moses et al., 2003). Although these strategies significantly reduced restenosis rates, clinical and pathological investigations revealed a delicate balance between suppression of smooth muscle proliferation and preservation of endothelial repair (Joner et al., 2006).

Incomplete reendothelialization and impaired arterial healing were associated with increased vulnerability to late and very late thrombotic events, particularly in the setting of premature discontinuation of antiplatelet therapy (Iakovou et al., 2025). Subsequent refinements in stent architecture—including thinner struts, improved alloy composition, and more biocompatible or biodegradable polymer coatings—demonstrated improved safety profiles in large, randomized trials (Stone et al., 2010). Nevertheless, accumulating pathological evidence indicates that durable polymer coatings and metallic scaffolds may continue to provoke chronic inflammatory responses and delayed vascular healing in susceptible contexts (Finn et al., 2007a; Virmani et al., 2004).

These observations highlight a fundamental paradigm shift in stent development: from purely mechanical revascularization devices toward biologically integrated platforms that actively modulate vascular healing, inflammation, and thrombogenicity (Palmerini et al., 2013). Importantly, while much of the mechanistic understanding of stent failure has been derived from coronary experience, intracranial arteries possess distinct anatomical, hemodynamic, and immunological characteristics. Direct extrapolation from coronary paradigms to neurovascular applications may therefore be inappropriate. A mechanistic framework integrating endothelial repair imbalance, smooth muscle phenotypic modulation, lipid-driven remodeling, and thrombo-inflammatory amplification is essential for guiding the rational development of next-generation intracranial stents. A comparative overview of representative stent categories, material properties, and mechanism-related risk profiles is summarized in Table 1.

**TABLE 1:** Comparative overview of stent categories and mechanism-related risk profiles.

Deployment method	Therapeutic category	Representative devices	Primary materials/Design features	Major clinical indications	Mechanism-related risk profile
Self-expanding	Neuro-dedicated stents	Wingspan, enterprise, neuroform atlas, LVIS	Nitinol (laser-cut or braided); flexible, low radial force	Intracranial atherosclerotic disease; aneurysm neck bridging; adjunctive support in coil embolization	Altered wall shear stress; thin medial layer; NVU-related endothelial vulnerability; delayed endothelialization
	Flow-diverting stents	Pipeline, tubridge	High metal coverage nitinol mesh; dense braided structure	Large or giant IAs	High metal surface area; disturbed flow remodeling; delayed endothelial healing; thrombo-inflammatory activation
Balloon-expandable	Coronary bare-metal stents (BMS)	Multi-link (abbott)	Stainless steel or cobalt–chromium alloy scaffold	Coronary artery disease (early-generation revascularization)	Elastic recoil; neointimal hyperplasia; early platelet-driven thrombo-inflammatory activation
	Coronary drug-eluting stents (DES)	Cypher, xience	Metallic scaffold + antiproliferative drug (sirolimus, everolimus, paclitaxel) + polymer coating	Prevention of coronary ISR	Delayed re-endothelialization; polymer hypersensitivity; chronic inflammation; neoatherosclerosis; late thrombotic risk
	Intracranial balloon-expandable stents	Apollo	Stainless steel or cobalt–chromium balloon-expandable design	Selected symptomatic intracranial stenosis	Higher radial force; vessel wall injury; endothelial denudation; restenosis risk
	Covered stents	Willis stent, graftmaster	PTFE or ePTFE membrane + metallic scaffold	Pseudoaneurysm; vessel rupture; carotid-cavernous fistula	High thrombogenicity of synthetic membrane; delayed endothelialization; side-branch occlusion; altered local flow patterns

## 3 Molecular and cellular mechanisms of ISR and stent-associated thrombosis

### 3.1 Endothelial injury spectrum and repair imbalance

Endothelial injury following stent deployment represents a dynamic spectrum of structural disruption, regulated cell death, and functional impairment rather than a single denudation event. Under physiological conditions, endothelial cells maintain vascular homeostasis through barrier integrity, anti-inflammatory signaling, and release of antithrombotic mediators including nitric oxide, prostacyclin, thrombomodulin, and tissue plasminogen activator (Mehta and Malik, 2006; Simionescu and Antohe, 2006; Jimenez and Davies, 2009; Xu and Zou, 2009; Wu and Thiagarajan, 1996). Mechanical expansion of a stent directly disrupts this protective interface, exposing subendothelial collagen and triggering platelet adhesion and activation of the coagulation cascade (Todorov et al., 2021). Beyond immediate mechanical denudation, endothelial cell loss after stent implantation involves distinct forms of cell death directly associated with stent-induced injury.

First, mechanical stretch and barotrauma exerted by expanding stent struts induce cytoskeletal disruption and mitochondrial stress, activating caspase-dependent apoptotic pathways in endothelial cells (Durand et al., 2004; Farb et al., 1999). Histopathological analyses of human coronary stents have demonstrated endothelial apoptosis adjacent to stent struts during the early post-implantation phase, linking mechanical injury to thrombogenic surface exposure and acute thrombus formation (Farb et al., 1999). Second, in regions of excessive oxidative stress generated by disturbed shear stress and platelet-driven inflammatory activation after stenting, reactive oxygen species (ROS) accumulation may exceed endogenous antioxidant buffering capacity. Severe oxidative stress can result in loss of membrane integrity and necrotic endothelial injury, thereby amplifying inflammatory signaling and tissue factor–mediated coagulation activation (Witztum and Steinberg, 1991). This necrotic damage further enhances thrombo-inflammatory propagation within the stented segment. Third, ferroptosis—an iron-dependent and lipid peroxidation–driven form of regulated cell death—has emerged as a mechanistic link between redox imbalance and endothelial dysfunction (Stockwell et al., 2017; Li et al., 2020). Ferroptosis is characterized by accumulation of lipid peroxides and iron-dependent oxidative injury (Stockwell et al., 2017). Although direct evidence specifically demonstrating ferroptosis in intracranial stenting remains limited, cerebral endothelial cells exhibit heightened susceptibility to iron-mediated oxidative stress (Stockwell et al., 2017; Carocci et al., 2018). In the context of stent-induced disturbed flow and oxidative microenvironmental changes, ferroptotic mechanisms may contribute to impaired endothelial regenerative capacity, particularly within the neurovascular unit.

In parallel with direct cell death pathways, stent-induced geometric alteration substantially modifies endothelial phenotype through disturbed shear stress patterns. Regions of low and oscillatory shear stress promote proinflammatory transcriptional programming and reduce nitric oxide bioavailability (Otsuka et al., 2015; Chiu and Chien, 2011). Pathological studies demonstrate that incomplete endothelial coverage and persistent fibrin deposition constitute major substrates for late stent thrombosis (Joner et al., 2006; Finn et al., 2007a). Thus, endothelial dysfunction after stent implantation reflects an integrated process in which mechanical trauma, apoptosis, oxidative necrosis, ferroptosis-related redox imbalance, and hemodynamic disturbance converge to delay re-endothelialization. Importantly, endothelial repair capacity differs across vascular territories. In intracranial arteries, regeneration occurs within the tightly regulated neurovascular unit (NVU), where endothelial cells interact with pericytes, astrocytes, and microglia to maintain blood–brain barrier integrity (Zlokovic, 2008; Sweeney et al., 2018; Armulik et al., 2010). Cerebral endothelial cells demonstrate increased vulnerability to oxidative and iron-mediated injury (Stockwell et al., 2017; Armulik et al., 2010), potentially contributing to delayed endothelial recovery and increased thrombotic susceptibility following intracranial stent implantation compared with coronary vessels (Chimowitz et al., 2011; Gao et al., 2022). These vascular bed–specific constraints underscore the importance of endothelial preservation as a central determinant of long-term stent healing.

### 3.2 Vascular smooth muscle cell phenotypic modulation and structural remodeling

While endothelial injury initiates thrombotic signaling, vascular smooth muscle cell (VSMC) phenotypic modulation drives structural remodeling within the stented segment. Under physiological conditions, VSMCs maintain a contractile phenotype that preserves vascular tone and structural stability (Owens et al., 2004). Following vascular injury, growth factors such as platelet-derived growth factor and inflammatory mediators promote transition toward a synthetic phenotype characterized by enhanced proliferation, migration, and extracellular matrix production (Khachigian et al., 2022; Deglise et al., 2022).

This phenotypic switch underlies neointimal hyperplasia, the principal substrate of early ISR. Experimental studies demonstrate that VSMC activation is temporally dynamic, with peak migratory signaling occurring during the early post-injury phase (Huang et al., 2019). In addition to resident VSMCs, circulating progenitor cells and vascular stem cell populations may contribute to neointimal formation (Hibbert et al., 2003; Hibbert et al., 2004). Single-cell transcriptomic analyses further reveal heterogeneity among VSMC subsets in injured vessels, identifying proliferative and inflammatory clusters regulated by enhancer networks and transcriptional modulators (Lambert et al., 2024).

Thus, VSMC-driven phenotypic switching and proliferation constitute the primary structural arm of early stent failure. Meta-analytic data in intracranial atherosclerotic stenosis further confirm the clinical relevance of this early VSMC-driven ISR after stenting, underscoring the time-dependent nature of cerebral vascular remodeling (Peng et al., 2020).

### 3.3 Inflammatory and immune amplification

Inflammatory activation and immune cell recruitment permeate the pathological course of ISR and stent-associated thrombosis, functioning in concert with coagulation activation to sustain thrombo-inflammatory signaling. Early leukocyte recruitment to adherent platelets influences neointimal formation, as demonstrated by reduced hyperplasia in β3-integrin–deficient models (Smyth et al., 2001). Stents, as implanted biomaterials, may provoke localized hypersensitivity responses involving eosinophil infiltration and delayed inflammatory reactions (Virmani et al., 2004; Chen, 2008; Hillen et al., 2002).

Complement activation further links innate immunity to thrombosis. Activation of classical, lectin, and alternative complement pathways generates C3a, C5a, and the membrane attack complex (C5b-9), which enhance platelet activation, endothelial adhesion molecule expression, and tissue factor activity (Rawish et al., 2021; Oikonomopoulou et al., 2012; Tedesco et al., 1997; Ricklin et al., 2010). Complement–coagulation crosstalk thereby establishes a self-reinforcing thrombo-inflammatory circuit.

Phenotypically modulated VSMCs also contribute to inflammatory amplification. MicroRNA-21 signaling promotes neointimal formation (McDonald et al., 2015), while inflammatory mediators such as CTRP5 enhance VSMC proliferation and migration through Notch and TGF-β–dependent pathways (Shen et al., 2017). Circulating inflammatory biomarkers including high-sensitivity C-reactive protein and Rap1A correlate with ISR risk (Gao et al., 2023).

In intracranial vessels, inflammatory amplification may be further modulated by neurovascular unit signaling and blood–brain barrier perturbation, potentially intensifying local immune–vascular coupling after stent implantation. Collectively, immune activation represents the amplification arm of stent pathology, integrating endothelial injury and structural remodeling into a persistent thrombo-inflammatory state.

### 3.4 Neoatherosclerosis: the chronic inflammatory substrate for late stent failure

While early ISR is primarily driven by VSMC proliferation, late stent failure is frequently mediated by neoatherosclerosis, a common pathological substrate for both ISR and in-stent thrombosis (IST). Rather than simple structural remodeling, neoatherosclerosis can be conceptualized as a chronic inflammatory remodeling process that evolves from the initial vascular injury response and is perpetuated by persistent stimulation from stent struts and eluted drugs (Otsuka et al., 2015; Nakazawa et al., 2011). It is characterized by lipid accumulation, macrophage infiltration, necrotic core formation, and calcification within the neointima. Persistent endothelial dysfunction, alongside the chronic foreign-body response to durable polymers or metallic scaffolds, accelerates this transformation. This chronic inflammatory milieu not only drives progressive luminal renarrowing (late ISR) but also establishes a highly prothrombotic substrate. Upon plaque rupture or endothelial erosion, the lipid-rich necrotic core is exposed to circulating blood, precipitating late or very late stent thrombosis (Joner et al., 2006; Finn et al., 2007b; Otsuka et al., 2012).

## 4 Thrombo-inflammatory amplification and hemodynamic modulation

While endothelial disruption initiates thrombus formation, subsequent thrombo-inflammatory amplification determines the persistence and propagation of stent-associated thrombosis. Beyond early coagulation activation, platelet-driven signaling, complement cascades, metabolic factors, and hemodynamic perturbations interact to create a self-reinforcing pathological microenvironment. These mechanisms are particularly relevant in intracranial arteries, where unique vascular structure and neurovascular unit regulation may influence post-stenting healing dynamics.

### 4.1 Platelet-driven thrombo-inflammatory amplification

Platelet activation represents one of the earliest biological responses following stent implantation. Mechanical endothelial denudation and exposure of subendothelial collagen rapidly trigger platelet adhesion through vWF-dependent mechanisms, leading to aggregation and mural thrombus formation. Beyond their classical hemostatic function, platelets act as active immunomodulatory cells linking coagulation and inflammation (Farb et al., 1999; Farb et al., 2002; Tanguay et al., 2003).

Activated platelets release platelet-derived microvesicles (PMVs), which function as potent procoagulant and proinflammatory mediators. The externalized phosphatidylserine on PMVs provides a catalytic surface for prothrombinase complex assembly, thereby accelerating thrombin and fibrin generation (Owens and Mackman, 2011). In addition to amplifying coagulation, PMVs facilitate intercellular communication. Through P-selectin–dependent interactions, PMVs engage neutrophils and promote the formation of neutrophil extracellular traps (NETs), establishing a platelet–microvesicle–neutrophil amplification axis that enhances thrombus propagation (Fuchs et al., 2010).

At later stages, platelet- and vessel wall–derived microRNAs further influence vascular remodeling. MicroRNAs such as miR-221 and miR-222 regulate VSMC proliferation, endothelial signaling, and inflammatory pathways and have been implicated in neointimal progression (Liu et al., 2009). Thus, PMVs and microRNA-mediated signaling form an intermediate biological layer linking acute thrombus formation to subsequent structural remodeling. However, most mechanistic data derive from non–stent-specific vascular models, and definitive causal validation in intracranial stenting remains limited.

In the intracranial circulation, platelet activation may additionally interact with BBB perturbation and neuroinflammatory responses. Crosstalk between activated platelets and components of the neurovascular unit—including endothelial cells, pericytes, and astrocytic end-feet—may further amplify thrombo-inflammatory signaling within the confined cerebral vascular microenvironment.

### 4.2 Complement–coagulation crosstalk

The complement system serves as a critical interface between innate immunity and thrombosis, functioning as an amplifier rather than a linear effector pathway. Complement activation through classical, lectin, or alternative pathways generates the anaphylatoxins C3a and C5a, as well as the membrane attack complex (C5b-9), each exerting distinct effects on vascular and platelet biology (Rawish et al., 2021; Oikonomopoulou et al., 2012).

Experimental evidence suggests that C3a and C5a enhance platelet activation and aggregation through their respective receptors (C3aR and C5aR), promoting procoagulant surface exposure and reinforcing thrombin generation (Oikonomopoulou et al., 2012). In parallel, sublytic deposition of C5b-9 on endothelial cells induces oxidative stress and upregulation of adhesion molecules, thereby linking complement activation to endothelial dysfunction and leukocyte recruitment (Tedesco et al., 1997). This bidirectional crosstalk between complement and coagulation pathways contributes to a self-propagating thrombo-inflammatory circuit within the stented segment (Rawish et al., 2021).

Complement components also interact with the kallikrein–kinin system, modulating vascular permeability and inflammatory signaling (Bekassy et al., 2022). Although pharmacological inhibition of terminal complement activation (e.g., anti-C5 therapy) has demonstrated efficacy in thrombotic microangiopathies, systemic complement suppression increases susceptibility to infection. Therefore, spatially and temporally selective modulation may be more appropriate in the context of stent-associated thrombosis.

While most mechanistic evidence derives from coronary or systemic vascular models, complement-mediated endothelial activation may be particularly relevant in intracranial arteries, where BBB integrity and neuroimmune interactions introduce additional regulatory complexity. Targeting complement pathways in intracranial stenting thus requires careful consideration of vascular bed–specific immune–vascular coupling.

### 4.3 Hemodynamic and metabolic modulation of mechanosensitive thrombosis

Stent implantation inevitably alters local vascular geometry and shear stress distribution. Hemodynamic disturbances—particularly low and oscillatory shear stress between stent struts and the vessel wall—facilitate local accumulation of platelets, coagulation factors, and inflammatory cells (Nesbitt et al., 2009; Xue et al., 2020; Kolandaivelu et al., 2011). High shear stress promotes conformational unfolding of vWF, enhancing platelet tethering and aggregation (Nesbitt et al., 2009; Jackson, 2011), whereas low shear regions support sustained fibrin deposition and endothelial dysfunction (Joner et al., 2006; Finn et al., 2007a; Lib and by, 2002).

The temporal evolution of thrombosis following stent deployment reflects dynamic interaction between mechanical and biological factors. During the acute phase, shear-dependent platelet adhesion predominates. In the subacute phase, persistent disturbed flow promotes fibrin persistence and inflammatory cell infiltration. In the chronic phase, sustained oscillatory shear impairs endothelial nitric oxide signaling and accelerates neoatherosclerosis, a recognized substrate for late stent failure (Chiu and Chien, 2011; Nakazawa et al., 2011; Otsuka et al., 2014a).

Beyond mechanical factors, metabolic risk burden substantially modifies thrombotic susceptibility within stented segments. Apolipoprotein B–containing lipoproteins—including low-density lipoprotein (LDL), very-low-density lipoprotein (VLDL), and chylomicron remnants—exert direct effects on endothelial biology and coagulation pathways (B et al., 2020; Nordestgaard, 2016). Oxidized LDL (oxLDL) reduces nitric oxide bioavailability, upregulates adhesion molecules, and enhances tissue factor expression, thereby promoting thrombin generation and shifting the vascular surface toward a procoagulant phenotype (Lib and by, 2002; Steinberg, 1997; Badimon and Vilahur, 2012).

Disturbed-flow microenvironments created by stent struts favor subendothelial retention and oxidative modification of LDL and remnant lipoproteins (Chiu and Chien, 2011). Experimental evidence further demonstrates that oxLDL enhances platelet activation through CD36-dependent pathways and promotes platelet–leukocyte aggregate formation, thereby linking metabolic dysregulation to thrombo-inflammatory amplification (Flowers et al., 2025; Podrez et al., 2007). Within drug-eluting stents, delayed endothelialization may facilitate lipid infiltration into the developing neointima. Pathological analyses of neoatherosclerosis reveal accumulation of lipid-laden macrophages and necrotic core formation within stented segments, changes associated with late thrombotic events (Nakazawa et al., 2011; Otsuka et al., 2014a).

Collectively, these findings support a metabolism–flow–thrombosis axis in which systemic lipid burden interacts with stent-induced hemodynamic disturbance to amplify local thrombo-inflammatory signaling. In this framework, LDL, VLDL, and remnant lipoproteins are not merely background cardiovascular risk factors but active modulators of stent-associated thrombosis and late restenosis.

## 5 Vascular bed–specific pathobiological differences

### 5.1 Coronary in-stent restenosis: structural and hemodynamic determinants

Coronary ISR represents a multifactorial pathological process shaped by local plaque composition, vascular wall structure, systemic metabolic burden, and postprocedural hemodynamic conditions. In advanced coronary atherosclerosis, calcific remodeling and neointimal calcification frequently contribute to restenosis. Optical coherence tomography (OCT) studies have demonstrated that calcified nodules may protrude between stent struts, mechanically narrowing the lumen and predisposing to target lesion revascularization (Buccheri et al., 2016; Otsuka et al., 2014b). These findings underscore that structural plaque characteristics substantially influence ISR susceptibility.

Functional hemodynamic assessment has emerged as a critical adjunct to anatomical evaluation. Post-procedural indices such as fractional flow reserve (Steinberg, 1997) and quantitative flow ratio (QFR) are strongly associated with vessel-related adverse outcomes, including repeat revascularization (Pijls, 2013; Hwang et al., 2022; Liu W. et al., 2023). Suboptimal flow restoration immediately after stent deployment may act as a hemodynamic trigger for accelerated neointimal progression, emphasizing that long-term stent patency depends on both favorable plaque modification and optimized local shear conditions.

Genetic predisposition further modulates coronary ISR risk. Variants in inflammatory and vascular remodeling pathways have been associated with increased susceptibility to restenosis (Monraats et al., 2005). These observations suggest that coronary ISR reflects not only proliferative responses but also structural vulnerability, biomechanical stress distribution, and host-specific inflammatory reactivity.

Importantly, while coronary ISR has been extensively characterized, most mechanistic insights derive from systemic vascular biology, where collateral flow and robust adventitial support partially buffer adverse remodeling.

### 5.2 Intracranial arteries: neurovascular constraints on stent healing

In contrast to coronary arteries, intracranial vessels operate within the tightly regulated microenvironment of the NVU. The absence of an external elastic lamina, thinner medial smooth muscle layers, and limited adventitial support render intracranial arteries structurally distinct (Ritz et al., 2014). Moreover, endothelial integrity in cerebral vessels is intrinsically linked to BBB homeostasis, which depends on coordinated interactions among endothelial cells, pericytes, astrocytic end-feet, and microglia (Zlokovic, 2008; Sweeney et al., 2018; Armulik et al., 2010).

Following stent implantation, endothelial denudation in intracranial arteries not only disrupts luminal integrity but may also perturb BBB-specific junctional complexes. Unlike systemic vessels, where endothelial repair is largely governed by local vascular and circulating factors, cerebral endothelial regeneration occurs within an NVU-coupled framework. BBB disturbance can rapidly activate perivascular microglia and astrocytes, initiating localized neuroinflammatory signaling (Sweeney et al., 2018; Herz et al., 2017). Activated glial cells release interleukin-1β, tumor necrosis factor-α, and reactive oxygen species, which may impair endothelial nitric oxide bioavailability and delay re-endothelialization. This glial–endothelial feedback amplification may represent a distinctive mechanism contributing to persistent thrombo-inflammatory signaling in cerebral stented segments.

Pericytes play a central stabilizing role in maintaining BBB integrity and regulating endothelial phenotype. Experimental evidence demonstrates that pericyte deficiency leads to altered endothelial gene expression programs and increased permeability (Armulik et al., 2010). Given the thinner media and reduced adventitial buffering capacity of intracranial arteries, pericyte-mediated endothelial stabilization may be particularly critical after mechanical injury induced by stent deployment. Disruption of pericyte–endothelial coupling may therefore prolong endothelial vulnerability and compromise vascular repair capacity in the cerebral circulation.

Cerebral endothelial cells also exhibit heightened susceptibility to iron-mediated oxidative stress and ferroptosis. Ferroptosis, a regulated cell death process driven by lipid peroxidation, has been increasingly implicated in central nervous system injury models (Stockwell et al., 2017). In the context of intracranial stenting, mechanical trauma combined with altered microcirculatory flow and potential iron accumulation may amplify lipid peroxidation within endothelial cells, thereby impairing regenerative capacity. Such ferroptotic vulnerability may constitute a mechanistically distinct contributor to delayed endothelial healing in cerebral arteries, particularly in patients with coexisting small vessel disease or chronic hypoperfusion.

Hemodynamic factors further distinguish intracranial stent healing from coronary paradigms. Cerebral arteries operate within the closed collateral architecture of the Circle of Willis, where geometric alteration after stent deployment may redistribute flow across communicating segments. Intracranial computational fluid dynamics studies demonstrate that vascular geometry significantly influences local wall shear stress and oscillatory shear index patterns (Schirmer and Malek, 2008; Benndorf et al., 2009; Fujimoto et al., 2014; Cebral et al., 2011). These disturbed-flow conditions modulate endothelial mechanotransduction pathways, including shear-sensitive anti-inflammatory signaling cascades (Chiu and Chien, 2011), thereby contributing to spatially heterogeneous remodeling responses unique to cerebral vessels.

Collectively, intracranial stent healing should be conceptualized as an NVU-coupled repair process characterized by endothelial–glial–pericyte crosstalk, BBB-sensitive inflammatory amplification, ferroptotic susceptibility, and Circle of Willis–dependent hemodynamic redistribution. These mechanisms distinguish cerebral vascular repair from coronary paradigms and underscore the necessity of vascular bed–specific therapeutic strategies for next-generation intracranial stent platforms.

Intracranial stenting outcomes provide direct evidence that cerebral arteries exhibit vascular bed–specific healing and complication profiles. Randomized trials, including SAMMPRIS (Chimowitz et al., 2011) and VISSIT (Zaidat et al., 2015), reported higher early periprocedural stroke risk with stenting compared with aggressive medical therapy, highlighting the unique vulnerability of intracranial circulation during the acute healing phase. More recent data with refined on-label selection and experienced operators (WEAVE) (Alexander et al., 2019) and its 1-year follow-up (WOVEN) (Alexander et al., 2021) suggest that periprocedural risk can be substantially reduced, yet long-term events remain non-negligible. Consistent with these observations, a systematic review and meta-analysis of symptomatic ICAS after stenting reported a measurable incidence of in-stent restenosis with a proportion becoming symptomatic, underscoring that intracranial ISR is clinically relevant and may reflect distinct endothelial repair and remodeling constraints in cerebral arteries (Peng et al., 2020).

Histological studies indicate that vasa vasorum are relatively limited in intracranial arteries (with age- and disease-associated emergence), which may constrain adaptive remodeling and influence lesion biology compared with extracranial/coronary vessels (Takaba et al., 1998; Zheng et al., 2018).

### 5.3 Shared core mechanisms and divergent clinical implications

Despite anatomical and physiological differences, coronary and intracranial ISR share a common thrombo-inflammatory core. Endothelial injury, platelet activation, complement amplification, hemodynamic disturbance, and metabolic modulation interact to drive neointimal remodeling and thrombotic risk (Farb et al., 1999; Chiu and Chien, 2011). However, the clinical consequences of stent failure differ markedly across vascular beds.

In coronary circulation, collateral vessels may partially mitigate ischemic injury (Seiler et al., 2013). In contrast, intracranial arteries function as terminal vessels with limited compensatory flow. Consequently, even minor restenosis or thrombosis may directly translate into ischemic stroke. Moreover, BBB disruption and NVU involvement introduce additional layers of immune–vascular coupling does not present in systemic arteries.

These distinctions have important translational implications. While coronary stent development has traditionally focused on suppressing smooth muscle proliferation, next-generation intracranial stent platforms may require a more nuanced approach—balancing antiproliferative efficacy with preservation of endothelial integrity, modulation of thrombo-inflammatory amplification, and maintenance of physiologic shear stress patterns.

**FIGURE 1:**
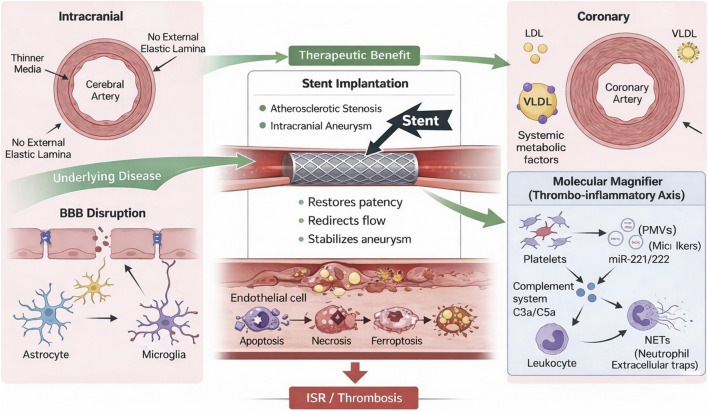
Integrated mechanistic framework of thrombo-inflammatory remodeling in coronary and intracranial stent healing.

Future intracranial stent design should therefore integrate biological compatibility, hemodynamic optimization, and spatially selective immune modulation, rather than relying solely on cytostatic drug delivery. A vascular bed–specific strategy is essential to improve long-term patency while minimizing catastrophic cerebrovascular events. The integrated mechanistic framework proposed in this review is schematically illustrated in Figure 1, and key structural, biological, and hemodynamic distinctions between coronary and intracranial stent healing are summarized in Table 2.

**TABLE 2:** Key biological and hemodynamic differences between coronary and intracranial stent healing.

Domain	Coronary arteries	Intracranial arteries
External elastic lamina	Present	Absent
Medial thickness	Thick	Thin
Vasa vasorum	Abundant	Limited
BBB coupling	No	Yes (NVU-dependent)
Flow dynamics	Segmental	Circle of willis redistribution
ISR pattern	Early hyperplasia dominant	Variable, NVU-influenced
Thrombotic risk profile	Delayed healing (DES)	Early hazard + ISR variability

## 6 Conclusions and future perspectives

Stent-associated restenosis and thrombosis should no longer be viewed as isolated mechanical complications, but as dynamic thrombo-inflammatory remodeling processes shaped by endothelial injury, platelet amplification, complement activation, hemodynamic perturbation, and metabolic modulation. Across vascular territories, these biological layers interact to determine long-term vascular healing and device durability.

While coronary stent pathology has been extensively characterized, direct extrapolation to intracranial arteries requires caution. Cerebral vessels differ fundamentally in structural architecture, blood–brain barrier integration, collateral flow dynamics, and neuroimmune regulation. NVU disruption, circle of Willis–dependent flow redistribution, and heightened endothelial vulnerability collectively predispose intracranial stented segments to distinct thrombo-inflammatory amplification and repair imbalance.

Contemporary understanding of ISR extends beyond neointimal hyperplasia to include neoatherosclerosis, persistent endothelial dysfunction, and chronic feedback between coagulation and inflammation. Accordingly, next-generation intracranial stent development should adopt mechanism-based design strategies rather than single-pathway suppression. Endothelial-preserving approaches—such as nitric oxide–releasing surfaces, endothelial progenitor cell–capturing coatings, or redox-modulating platforms—may accelerate re-endothelialization while minimizing thrombosis. Localized anti-inflammatory or complement-modulating coatings could attenuate thrombo-inflammatory amplification, particularly within NVU-coupled cerebral circulation. In parallel, hemodynamically optimized stent architectures guided by patient-specific computational fluid dynamics may reduce disturbed-flow–induced remodeling.

Integration of high-resolution vessel wall imaging, molecular profiling, and bioengineering innovation will be essential to enable individualized, vascular bed–specific intracranial stent strategies. Ultimately, transitioning from purely mechanical revascularization toward biologically adaptive platforms represents a critical step toward durable patency and improved cerebrovascular outcomes.
